# Development of addiction to nicotine with patients suffering from schizophrenia

**DOI:** 10.1192/j.eurpsy.2025.2227

**Published:** 2025-08-26

**Authors:** M. Wadoń, K. Krysta

**Affiliations:** 1 Department of Rehabilitaction Psychiatry, Medical University of Silesia, Katowice, Poland

## Abstract

**Introduction:**

Symptoms of schizophrenia are associated with dysregulation of the dopaminergic systems of the central nervous system. Negative symptoms, including those related to emotions and mood, result from reduced activity in the mesocortical system, while positive symptoms result from increased activity of the mesolimbic system. Disorders in the reward system, composed of dopaminergic neurons, play an important role in the pathogenesis of addiction development. Nicotine activates the presynaptic parts of the neurons that make up the system to secrete dopamine, which translates into a feeling of pleasure and stimulates the development of addiction.

**Objectives:**

The aim of the study was to demonstrate the relationship between the degree of development of nicotine and the severity of symptoms related to emotions and mood with patients suffering from schizophrenia.

**Methods:**

The study involved 75 patients diagnosed with schizophrenia (F.20 according to ICD-10). The study was conducted from October 2023 to September 2024 in CZPiLU in Gliwice. During the study, the Frankfurter-Bejindlichkeits-Skala was used to assess the appearance and severity of symptoms related to emotions and mood. The analysis of the development of addiction was made possible by the Fagerström Test for Nicotine Dependence.

**Results:**

Based on statistical analysis, a significant relationship was noted between the severity of addiction to nicotine and the presence or absence of symptoms related to well-being. In addition, a correlation (p<0,001) was noted between the degree of nicotine and the severity of symptoms related to emotions and mood. Patients with strong negative symptoms of schizophrenia developed a high level of addiction to nicotine (p=0,001), while those with mild (p=0,001) or moderate symptoms (p=0,005) related to well-being achieved lower scores on individual addiction scales. All patients included in the study had at least a mild level of addiction to nicotine.

**Conclusions:**

The presence of schizophrenia symptoms related to well-being has a significant impact on the initiation of addiction development to nicotine. Furthermore, the level of advancement of symptoms related to emotions and mood translates into the severity of nicotine, especially in the group of patients in whom strong negative symptoms translate into a high level of addiction advancement. Patients suffering from schizophrenia require psychological and psychiatric care and diagnostics for addictions and appropriate prevention before their development.

**Disclosure of Interest:**

None Declared

